# Supplementary material for: Towards inclusive authorship: Analyzing author representation in PLOS Global Public Health front matter content
Source: PLOS Glob Public Health. 2025 Aug 18;5(8):e0005066. doi: 10.1371/journal.pgph.0005066 (PMC12360556; doi:10.1371/journal.pgph.0005066)
Supplement: S1 Table — (DOCX) [file pgph.0005066.s001.docx]

Supplementary File 1: Four main subject matter categories and included topics by category

| **Theme** | **Topics included** |
| --- | --- |
| Infectious diseases | *Nipah virus, mpox, Ebola, tuberculosis, malaria, polio, dengue, HIV, post-caesarean infection, COVID-19, neglected tropical diseases* |
| Non-communicable diseases (NCDs) | *Market-driven epidemics (overconsumption of sugar, cigarettes, and opioids), acute myocardial infarction, food insecurity, disability, injury, cancer, malnutrition, mental health issues* |
| Health infrastructure and delivery | *Wastewater management, sexual violence in healthcare delivery, pathogen surveillance systems, heatwave interventions, community health workers program, vaccine manufacturing, vaccine access, health financing assistance, WHO governance and delivery, global surgery, mental health infrastructure, climate change and health infrastructure, diagnostics, indication-based metrics in caesarean section rates, AI infrastructure access, healthcare system in Afghanistan, global health law reform, preventative policy, patient mobilisation, brain drain of nurses, dementia and ageing policy, visa and passport inequalities, human papillomavirus testing systems, work culture for midwives, Indigenous climate solutions, abortion care provision, universal health coverage, abuse in maternity care provision, pandemic preparedness infrastructure, decolonising humanitarian aid delivery, national health system ownership* |
| Research Partnerships | *Equitable long-term research partnerships, technology transfer and intellectual property, Indigenous knowledge in climate and nutrition research, Forum HIV Recency Assay Working Group, global therapeutics development coalition, Indigenous Knowledge Systems, inclusion of diverse voices in global health research, community engagement in research, language in global health partnerships, DEI research in health, global health research funders, digital technology for mental health research, single-issue advocacy, hierarchy and expertise in research and partnerships, healthy diet research, Global North-South partnerships, global health academia, bilateral research partnerships, paediatric research, equity in Canadian global health partnerships, use of race, ethnicity, and ancestry data in health research, Indigenous self-determination in health research, Nigerian perspective on global health research, reflexivity statements in research, global health research as activism, cooperation and research partnerships for equitable vaccination access* |
