# Supplementary material for: Towards inclusive authorship: Analyzing author representation in PLOS Global Public Health front matter content
Source: PLOS Glob Public Health. 2025 Aug 18;5(8):e0005066. doi: 10.1371/journal.pgph.0005066 (PMC12360556; doi:10.1371/journal.pgph.0005066)
Supplement: Attachment — Submitted filename: Peer Review Report - Meher Suri .pdf [file pgph.0005066.s003.pdf]

Peer Review Report

Reviewer: Meher Suri

1. Summary of the Research

This manuscript, *"Towards inclusive authorship: Analyzing author representation in PLOS Global Public Health front matter content,"* investigates the extent to which PLOS Global Public Health's commissioned content (including opinion pieces, reviews, and essays) has succeeded in reflecting equitable and diverse authorship, aligned with the journal's stated commitments to equity and decolonization. Using publicly available data on 878 authors across 136 articles published between October 2021 and December 2024, the authors systematically examine representation by World Bank country income category, gender, and Indigeneity. The study also analyzes the thematic and geographic focus of the articles and whether there is alignment between authorship and the subject matter of the content.

The results highlight substantial underrepresentation of Global South–affiliated authors, Indigenous authors, and nonbinary or gender-diverse individuals, with high-income country–affiliated authors comprising 69% of all contributors. Although articles focused on the Global South did show better representation of Global South authors (43% on average), overall the pattern remains one of inequity, especially in first and senior authorship positions. The authors recommend stronger and more proactive commissioning strategies to bring in historically marginalized voices.

This is an important and timely contribution to the literature on equity and epistemic justice in global health publishing, aligning with ongoing calls for decolonizing knowledge production. The work is clearly written, systematically designed, and includes actionable recommendations with direct relevance for editorial practice.

Strengths:

- Addresses a highly relevant and contemporary issue within global health scholarship.
- Methodologically systematic and transparent in its data collection and analysis approach.
- Contributes practical, actionable recommendations for journal editors and publishers.
- Well-aligned with the mission and values of PLOS Global Public Health.

Weaknesses:

- Reliance on publicly available data may obscure hidden or self-suppressed identities (e.g., non-disclosed Indigeneity, disability, or nonbinary gender).

- Use of institutional affiliation as a proxy for Global North/South status may oversimplify complex positionalities and lived experiences.

- Lacks deeper intersectional or qualitative analysis that might illuminate the interplay of multiple identities (e.g., how gender, country affiliation, and academic seniority interact).

Overall recommendation: *Accept with minor revisions.* The manuscript makes a valuable contribution and is highly relevant for the readership of *PLOS Global Public Health* and beyond. Addressing a few clarifications and expanding on conceptual points would further strengthen its rigor and impact.

2. Examples and Evidence

Major issues

1. **Critical discussion of Global North/South categorization**
   The authors use the World Bank income classification as a proxy for Global North/Global South, which is common in such analyses. However, this approach risks flattening the heterogeneity of author identities, particularly for scholars with lived experience of the Global South but based in HIC institutions. A more reflexive discussion of these limitations would be valuable to avoid essentializing identities by affiliation alone.

2. **Intersectionality and layered marginalizations**
   The paper currently describes gender, income affiliation, and Indigeneity separately. Yet intersectional scholarship shows these identities are interdependent and produce compounded marginalizations. While the authors note data limitations, a short reflective section on the significance of intersectionality even if only conceptually would enrich the discussion and acknowledge the layered nature of structural exclusion.

Minor issues

1. In the methods, the treatment of authors with multiple affiliations needs clearer explanation. For example, how were authors with a primary HIC affiliation but a secondary LMIC affiliation categorized? Please specify how these dual affiliations were systematically handled.

2. The discussion could briefly acknowledge language barriers and English-language hegemony in academic publishing, which is closely tied to epistemic injustice and may shape patterns of

authorship.

3. Minor consistency edits: there are a few inconsistent uses of hyphens (e.g., “Global-South-affiliated” vs. “Global South affiliated”) that should be standardized.

4. It may be helpful to mention the limited data on authors with disabilities earlier in the discussion, as this signals an area for future research and aligns with the authors’ broader equity framing.

3. Other Points

- I do not have any ethical concerns about this manuscript, given its use of publicly available data.

- I appreciate the transparent and reflexive nature of the work, which strengthens its ethical standing.

- I would be willing to review a revised version if needed.

Overall, this is a thoughtful, timely, and well-constructed study with high relevance to the field. I commend the authors for addressing epistemic justice within their own publishing ecosystem and hope the final revisions will strengthen the nuance and reflexivity of their analysis.
