# Supplementary material for: Towards inclusive authorship: Analyzing author representation in PLOS Global Public Health front matter content
Source: PLOS Glob Public Health. 2025 Aug 18;5(8):e0005066. doi: 10.1371/journal.pgph.0005066 (PMC12360556; doi:10.1371/journal.pgph.0005066)
Supplement: Attachment — Submitted filename: Review.docx [file pgph.0005066.s002.docx]

**Review**

Thank you for the opportunity to review “Towards inclusive authorship: Analyzing author representation in PLOS Global Public Health front matter content”. This is really important work! Below are some suggestions to help clarify arguments.

**MAJOR COMMENTS:**

- Your data is from 2021-2024 and you noted that PLOS GPH had a 2-year review in which the editorial board committed to being more intentional about including diverse authorship. Perhaps consider comparing author affiliation income categories in 2021-2022 vs. 2023-2024 to see if proportion of Global South authorship increased, using a basic statistical test. This could help inform whether more can be done to promote Global South authorship.

**MINOR COMMENTS:**

**Abstract:**

- Change “It has contributed to epistemic injustice” to “This has contributed to epistemic injustice”
- Where you say “has had an impact on policymaking” - do you mean policymaking as a downstream result of studies published in PLOS GPH?
- In the first instance of “PLOS Global Public Health” put in brackets beside it “(GPH)”
- Remove the comma in “PLOS Global Public Health, was launched in 2021”
- Change “using [genderize.io](http://genderize.io).” to “used [genderize.io](http://genderize.io).”
- “and when unavailable, using [genderize.io](http://genderize.io).” - in what percent of instances was it unavailable?

**Introduction:**

- Change “have been a long-standing challenge” to “have been long-standing challenges”
- Change “lack of journal policies on inclusive authorship, lack of support” to “lack of journal policies on inclusive authorship, **and** lack of support”
- Change “Epistemic injustice in global health knowledge production in turn influence global and national policies” to “Epistemic injustice in global health knowledge production, in turn, influences global and national policies”
- Change “Health Policy and Planning committing to at least 50% of articles include a first or senior author from a low and middle income country” to “Health Policy and Planning committed to including at least 50% of articles with a first or senior author from a low and middle income country”
- Change “In its two-year update, PLOS GPH editorial board” to “In its two-year update, PLOS GPH’s editorial board”

**Methods:**

- “Under authorship representation analysis we examined the country income category, gender, and Indigeneity of each author.” - How was group/consortium authorship dealt with?
- Change “we examined the country or region the article content is focused on” to “we examined the country or region the article content was focused on”
- “For first and senior authors, who often lead the writing and framing of commissioned opinion, review and essay articles we conducted an additional layer of analysis.” - after this sentence, you repeat the fact that you extract information on primary affiliations. I suggest removing that repeated information, and instead following this sentence with “In addition to primary institutional affiliations, for first and senior authors, we also examined the secondary ….”
- Change “Using the collected authorship data we did two types of calculations:” to “Using the collected authorship data we performed two different calculations:”
- By “b) the average of authorship based on country income category” do you mean “b) the average proportion of authorship based on country income category”?
- For gender and Indigeneity you note that you searched publicly available profiles - I suggest listing the exact online platforms that were searched, for reproducibility purposes. If there are many, perhaps include them in a supplementary table.
- Change “Supplementary File 1includes” to “Supplementary File 1 includes”

**Results:**

- Change “We considered all the authors (878), across all article types (136)” to “We considered all authors (n = 878) and articles (n = 136) in the following analyses.”
- Change “First (135) and senior (117) author represented across all commissioned articles” to “First (n = 135) and senior (n = 117) authors represented across all commissioned articles”
- Not sure if Table 3 is needed since all of the information is quite easy to summarise in text, as done in the paragraph above.
- “Using publicly available data and genderize.io we inferred gender identities across all 878 authors.” - for how many authors did you use [genderize.io](http://genderize.io)?
- Change “6 of the 117 senior authors (5%) self-identified as being Indigenous.” to “Six of the 117 senior authors (5%) self-identified as being Indigenous.”
- Be consistent with the use of “non-communicable diseases” vs NCDs - I suggest using the full-form in the first instance you mention it, then using NCDs afterwards.

**Discussion:**

- Change “PLOS GPH as of November 2023 had 50-50 Global South – Global North representation” to “As of November 2023, PLOS GPH had equal Global South and Global North representation”
- “HIC institution affiliated authors representation is much higher than non-HIC authors as well (Figure 4).” - do you mean Figure 1?
- Change “Therefore, in PLOS GPH front matter content about the Global South” to ”Therefore, PLOS GPH front matter content about the Global South”
- Remove “across all authors” from “Gender representation among commissioned content indicated higher representation of authors identifying as men versus women across all authors”
- Change “there is an opportunity for global health journal to platform views” to “there is an opportunity for global health journals to platform views”
- Under limitations - if the proportion of authors that required the use of [genderize.io](http://genderize.io) was high, this could also be a limitation
