# Supplementary material for: Towards inclusive authorship: Analyzing author representation in PLOS Global Public Health front matter content
Source: PLOS Glob Public Health. 2025 Aug 18;5(8):e0005066. doi: 10.1371/journal.pgph.0005066 (PMC12360556; doi:10.1371/journal.pgph.0005066)
Supplement: Attachment — Submitted filename: Revision Response Letter_July 22.docx [file pgph.0005066.s005.docx]

July 22, 2025

Dear Editorial Team,

Revised version of the *Towards inclusive authorship: Analyzing author representation in PLOS Global Public Health front matter content*

Thank you for the opportunity to strengthen our article with your feedback. We appreciate the thoughtful and detailed feedback. We did a careful evaluation of all comments and have made changes using the guidance offered. We have detailed the changes we made within the response table below and have provided rationale for our approach to this paper, where necessary. We have also added additional citations where necessary as well.

We look forward to the next steps in moving this towards publication. Please find the reviewer response table at the end of this letter.

Shashika Bandara

On behalf of the authorship team

| Reviewer 2 | |
| --- | --- |
| **Comments** | **Edits completed** |
| *Major comments* |  |
| Your data is from 2021-2024 and you noted that PLOS GPH had a 2-year review in which the editorial board committed to being more intentional about including diverse authorship. Perhaps consider comparing author affiliation income categories in 2021-2022 vs. 2023-2024 to see if proportion of Global South authorship increased, using a basic statistical test. This could help inform whether more can be done to promote Global South authorship. | Thank you for this thoughtful suggestion. We met as a team to discuss this approach.  We decided not to conduct this additional analysis since data is limited in the starting stages of the journal with fewer articles being submitted. For example, in 2021, there were only 6 articles published, and a further 31 in 2022, which would make the data sample size for this 2021-2022 period 37 articles, which we consider to be insufficient for meaningful comparison analysis. Another variable is the type of articles, which were solely opinion and review until Essay articles were introduced newly in 2024. Furthermore, our goal was to provide this analysis as a starting point for further conversations and – hopefully – analysis regarding authorship representation in global public health journals. We might consider conducting further analysis after a further five years or so and/or welcome another analysis of the progress made. We appreciate this suggestion and will keep in mind for future analyses. |
| *Abstract* |  |
| Change “It has contributed to epistemic injustice” to “This has contributed to epistemic injustice” | Thank you for this suggestion. We have incorporated it. |
| Where you say “has had an impact on policymaking” - do you mean policymaking as a downstream result of studies published in PLOS GPH? | Thanks for this comment. We meant lack of inclusion in all publications has had an impact on evidence informed policymaking. We have made an edit to clarify further. |
| In the first instance of “PLOS Global Public Health” put in brackets beside it “(GPH)” | Thank you for noting this. We have added the abbreviation. |
| Remove the comma in “PLOS Global Public Health, was launched in 2021” | Thank you for noting this. We have made this edit. |
| Change “using [genderize.io](http://genderize.io).” to “used [genderize.io](http://genderize.io).” | Thank you for noting this. We have made an edit to improve clarity. |
| “and when unavailable, using [genderize.io](http://genderize.io).” - in what percent of instances was it unavailable? | This is an important question. Thanks for raising this. As this is the abstract and has word limitations, we have described the approach in detail in the methods/results sections. |
| **Introduction** |  |
| Change “have been a long-standing challenge” to “have been long-standing challenges” | Thank you for the suggestion. We have incorporated the edit. |
| Change “Epistemic injustice in global health knowledge production in turn influence global and national policies” to “Epistemic injustice in global health knowledge production, in turn, influences global and national policies” | Thank you. We have added the suggested commas to the sentence to improve clarity. |
| Change “Health Policy and Planning committing to at least 50% of articles include a first or senior author from a low and middle income country” to “Health Policy and Planning committed to including at least 50% of articles with a first or senior author from a low and middle income country” | Thank you for noting this. We have made the edit. |
| Change “In its two-year update, PLOS GPH editorial board” to “In its two-year update, PLOS GPH’s editorial board” | Thank you for noting this. We have made the edit. |
| *Methods* |  |
| “Under authorship representation analysis we examined the country income category, gender, and Indigeneity of each author.” - How was group/consortium authorship dealt with? | Thank you for this thoughtful inquiry. We did not have group/consortium authorship in the articles we analyzed. Thus, we were able to analyze authorship representation using individual author data – which we explain in detail in the methods section. We have added a sentence to clarify that there were no group/consortium authorship. |
| Change “we examined the country or region the article content is focused on” to “we examined the country or region the article content was focused on” | Thank you for this suggestion. We have made the edit. |
| “For first and senior authors, who often lead the writing and framing of commissioned opinion, review and essay articles we conducted an additional layer of analysis.” - after this sentence, you repeat the fact that you extract information on primary affiliations. I suggest removing that repeated information, and instead following this sentence with “In addition to primary institutional affiliations, for first and senior authors, we also examined the secondary ….” | Thank you for this thoughtful suggestion. We have made the relevant edits. |
| Change “Using the collected authorship data we did two types of calculations:” to “Using the collected authorship data we performed two different calculations:” | Thank you for the suggestion. We have incorporated this edit. |
| By “b) the average of authorship based on country income category” do you mean “b) the average proportion of authorship based on country income category”? | Yes, thank you for noting this. We have made the correction. |
| For gender and Indigeneity you note that you searched publicly available profiles - I suggest listing the exact online platforms that were searched, for reproducibility purposes. If there are many, perhaps include them in a supplementary table. | Thank you for this suggestion to strengthen our methods reporting. We have included the main online sources we searched. |
| Change “Supplementary File 1includes” to “Supplementary File 1 includes” | Thank you for noting this. We have made the edit. |
| *Results* |  |
| Change “We considered all the authors (878), across all article types (136)” to “We considered all authors (n = 878) and articles (n = 136) in the following analyses.” | Thank you for the suggestion. We have made the edit. |
| Change “First (135) and senior (117) author represented across all commissioned articles” to “First (n = 135) and senior (n = 117) authors represented across all commissioned articles” | Thank you for the suggestion. We have made the edit. |
| Not sure if Table 3 is needed since all of the information is quite easy to summarise in text, as done in the paragraph above. | Thank for this suggestion. Since Tables helps digest information faster, we thought it would be helpful to still include despite the data being in the previous paragraph. |
| “Using publicly available data and genderize.io we inferred gender identities across all 878 authors.” - for how many authors did you use [genderize.io](http://genderize.io)? | Thank you for this suggestion – we have made edits across this paragraph and the ensuing ones to specify the number of authors, and the percentage of the total authors that we used the genderize.io tool to infer gender. |
| Change “6 of the 117 senior authors (5%) self-identified as being Indigenous.” to “Six of the 117 senior authors (5%) self-identified as being Indigenous.” | Thank you for this suggestion. We have made this edit and also made the corresponding edit in the previous sentence as well. |
| Be consistent with the use of “non-communicable diseases” vs NCDs - I suggest using the full-form in the first instance you mention it, then using NCDs afterwards. | Thanks for noting this. We have made the relevant edits. |
| *Discussion* |  |
| Change “PLOS GPH as of November 2023 had 50-50 Global South – Global North representation” to “As of November 2023, PLOS GPH had equal Global South and Global North representation” | Thank you for the suggestion. We have made this edit. |
| “HIC institution affiliated authors representation is much higher than non-HIC authors as well (Figure 4).” - do you mean Figure 1? | Thank you for noting this. We have corrected the reference to Figure 1. |
| Change “Therefore, in PLOS GPH front matter content about the Global South” to ”Therefore, PLOS GPH front matter content about the Global South” | Thank you for noting this. We have made the edit. |
| Remove “across all authors” from “Gender representation among commissioned content indicated higher representation of authors identifying as men versus women across all authors” | Thank you for this suggestion. Since we speak about first and senior author related gender representation in the following sentence, we thought retaining this detail to ensure clarity. |
| Change “there is an opportunity for global health journal to platform views” to “there is an opportunity for global health journals to platform views” | Thank you for noting this. We have made this edit. |
| Under limitations - if the proportion of authors that required the use of [genderize.io](http://genderize.io) was high, this could also be a limitation | Thank you for suggesting this to strengthen our limitations suggestion. The percentage of total authors that we used genderize.io for to infer the gender was low (8.4%). Thus, we decided not to include this as a key limitation, but chose to discuss the limitations of the genderize.io tool, to provide further context for reproducibility purposes. |
| Reviewer 3 | |
| **Comments** | **Edits Completed** |
| *Major Comments* |  |
| **Critical discussion of Global North/South categorization**  The authors use the World Bank income classification as a proxy for Global North/Global South, which is common in such analyses. However, this approach risks flattening the heterogeneity of author identities, particularly for scholars with lived experience of the Global South but based in HIC institutions. A more reflexive discussion of these limitations would be valuable to avoid essentializing identities by affiliation alone. | Thank you for this thoughtful suggestion. We have added text to highlight the points you raise in the discussion section. We also have cited relevant papers to support this point.  *“As scholars have argued before, we also recognize that the framing based on Global South versus Global North and World Bank country income categories can mask the complexity of identity, lived experiences, and interconnected representation of a country and are imperfect [32,33]. However, even with these limitations in categorization, as indicated above there is scope for strengthening representation.”* |
| **Intersectionality and layered marginalizations**  The paper currently describes gender, income affiliation, and Indigeneity separately. Yet intersectional scholarship shows these identities are interdependent and produce compounded marginalizations. While the authors note data limitations, a short reflective section on the significance of intersectionality even if only conceptually would enrich the discussion and acknowledge the layered nature of structural exclusion. | Thank you for this thoughtful suggestion. We have added text to highlight this point with a relevant citation.  *“Furthermore, intersectionality of identity is difficult to capture using secondary data. Often intersectionality of marginalized identities can compound the limitations of access and opportunity to publish. Therefore, we believe further research which examines authorship identity in an in-depth manner, including intersectionality of identity and its impact, is necessary [41].”* |
| *Minor Comments* |  |
| In the methods, the treatment of authors with multiple affiliations needs clearer explanation. For example, how were authors with a primary HIC affiliation but a secondary LMIC affiliation categorized? Please specify how these dual affiliations were systematically handled. | Thank you for this request for clarification.  We conducted primary author affiliations for all authors and conducted secondary affiliation analysis for first and senior authors. We have described the secondary author affiliation analysis separately in the methods section. Based on this comment, we added an additional clarification that it was only conducted for first and senior authors. Results are indicated in Figure 1 in addition to results reporting in text.  For reference, we have included the text from the methods section on secondary affiliation analysis here:  *“We also examined secondary institutional affiliations for first and senior authors. When secondary affiliations were considered, HIC representation saw marginal decrease. Among first authors, 97 of the 135 (72%) were affiliated exclusively with HICs, while 26 of 135 (19%) were affiliated exclusively with LICs, LMICs, and UMICs. A total of six of the 135 first authors (4%) listed dual affiliations with a primary HIC institution and a secondary LIC, LMIC, or UMIC institution, while another six of the 135 (4%) had primary LIC, LMIC, or UMIC affiliations with a secondary HIC institution (Figure 1).”* |
| The discussion could briefly acknowledge language barriers and English-language hegemony in academic publishing, which is closely tied to epistemic injustice and may shape patterns of authorship. | Thank you for this suggestion. We have incorporated this, and suggested language be a focus area in future research.  *An inter-related additional focus for future research could also be how language limitations (English being the sole language in many journals) impact academic publication access and opportunity, in turn affecting author representation.* |
| Minor consistency edits: there are a few inconsistent uses of hyphens (e.g., “Global-South-affiliated” vs. “Global South affiliated”) that should be standardized. | Thank you for noting this. We have made the relevant edits. |
| It may be helpful to mention the limited data on authors with disabilities earlier in the discussion, as this signals an area for future research and aligns with the authors’ broader equity framing. | Thank you for this suggestion. We considered altering the flow and structure. However, since we highlight the data limitation in a separate dedicated paragraph, we believe it does get sufficient attention – even though it is not mentioned earlier in the discussion. |
